# The role of exercise in reducing vasovagal syncope recurrence: A systematic review and meta-analysis

**DOI:** 10.1016/j.ajpc.2026.101504

**Published:** 2026-02-26

**Authors:** Amin Esmailian, Cyrus Raki, Hui Chen Han, Mohammad Alasti

**Affiliations:** a Department of Medicine, Peninsula University Hospital, 2 Hastings Rd, Frankston, Victoria 3199, Australia; b School of Clinical Sciences, Monash Health, Monash University, Melbourne, Victoria, Australia; c Victorian Heart Hospital, Clayton, Melbourne, Victoria, Australia; d Victorian Heart Institute, Monash University, Clayton, Melbourne, Victoria, Australia

**Keywords:** Vasovagal syncope, Exercise, Autonomic regulation, Systematic review and meta-analysis

## Abstract

**Background:**

Vasovagal syncope (VVS) is the most common cause of transient loss of consciousness, yet effective long-term treatments remain limited. Exercise has been proposed to improve autonomic regulation and lower recurrence rates. This study aims to determine the effect of structured exercise interventions on VVS recurrence and quality of life.

**Methods:**

This systematic review and meta-analysis were conducted according to PRISMA guidelines (PROSPERO CRD420251009841). MEDLINE, Embase, and Emcare were searched for studies published between 1 January 1998 and July 2025 for randomized and prospective interventional studies evaluating structured exercise in patients with VVS. Studies of non-exercise interventions or secondary causes of syncope were excluded. The primary outcome was recurrence of syncope; secondary outcomes included quality of life and physiological measures. Risk of bias was assessed using the Cochrane Collaboration Risk of Bias tool and Newcastle-Ottawa Scale. Random-effects meta-analysis was performed to pool recurrence proportions and mean differences with 95 % confidence intervals.

**Results:**

Eight studies (*n* = 435) were included (mean age range 21–39 years; 59 % female). The rate of VVS recurrence was lower in the intervention groups (7 %, 95 % CI 0.04–0.11) compared with control groups (24 %, 95 % CI 0.16–0.33). The pooled mean difference in syncopal events was –0.22 (95 % CI –0.42 to –0.02), indicating a statistically significant reduction in syncopal events for the exercise intervention compared with control groups. Quality-of-life scores improved by 30–45 points (*p* < 0.001), while modest, non-significant changes were observed in heart rate and blood pressure. Adherence exceeded 80 %, with no serious adverse events.

**Conclusion:**

Structured exercise was associated with lower VVS recurrence and improved quality of life. These findings are consistent with potential autonomic and cardiovascular adaptation. It represents a safe, effective, and sustainable adjunct to standard management. However, included studies were small and heterogenous and larger multicenter randomized trials are needed to confirm long-term efficacy.

**Lay summary:**

Structured physical exercise may help reduce fainting episodes in people with vasovagal syncope and improve their day-to-day confidence and wellbeing.

## Key findings

• Exercise-based programs were associated with fewer fainting recurrences compared with standard care alone.
• Across multiple studies, patients participating in structured exercise also reported meaningful improvements in functional status and quality of life.

**Figure:**
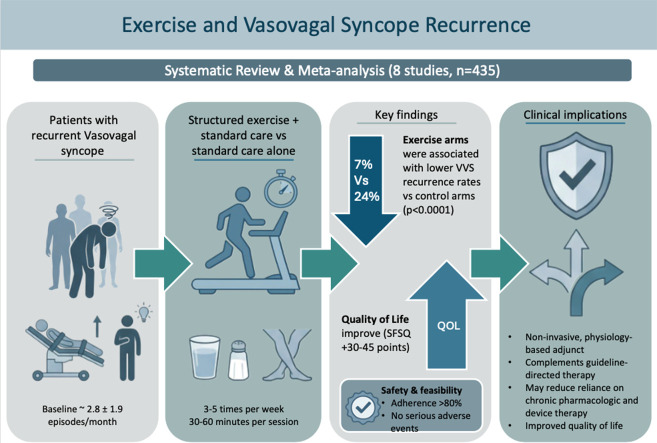
Central Illustration.

## Clinical implications

Structured exercise may serve as a beneficial adjunct in managing recurrent vasovagal syncope. The lower recurrence rates observed in exercise intervention groups, along with improvement in quality of life, may suggest a physiological benefit through enhanced autonomic regulation. While further large-scale studies are warranted, incorporating supervised or home-based exercise programs into individualized management plans could offer a safe and practical non-pharmacologic option for suitable patients.

## 1 Introduction

Vasovagal syncope (VVS) is the most common cause of transient loss of consciousness, affecting up to one-third of individuals during their lifetime [1]. Its clinical significance lies not in mortality, but in the risk of recurrent episodes leading to injury, impaired quality of life (QoL), and psychosocial distress [2].

Conventional management includes salt and fluid loading, physical counterpressure maneuvers, and pharmacological therapy with agents such as fludrocortisone, midodrine, or beta blockers [3,4]. While these strategies can reduce recurrence, their overall efficacy remains inconsistent, and pharmacological options are limited by side effects and adherence challenges [4].

Vasovagal syncope reflects a maladaptive reflex of cardiovascular autonomic control, typically triggered by orthostatic or emotional stress. In susceptible individuals, reduced ventricular filling with a concomitant rise in sympathetic tone and heart rate leads to vigorous contraction of an underfilled ventricle. This activates mechanosensitive and chemosensitive cardiac afferent C fibers, via receptors such as serotonin 5-HT_3_ and TRPV1, which project to medullary vasodepressor centers and precipitate abrupt withdrawal of sympathetic vasoconstrictor tone together with enhanced vagal efferent output [23]. The resulting bradycardia, peripheral vasodilation, and fall in cardiac output produce transient cerebral hypoperfusion and loss of consciousness [23]. Structured exercise training has been proposed as a non-pharmacological alternative. Endurance training can improve venous return, expand plasma volume, and enhance baroreflex sensitivity and orthostatic tolerance [5,6]. Regular endurance training may also influence central autonomic networks, improving sympathetic oscillatory amplitude and vagal-sympathetic coordination [[23], [24], [25]]. Yoga has also been investigated, combining physical conditioning with autonomic and stress modulation [[14], [15], [16]].

However, the effectiveness of exercise interventions across different regimens remains uncertain, with evidence limited to small randomized and observational studies. Although several studies have demonstrated potential benefits of structured exercise training in reducing vasovagal syncope recurrence, exercise is not currently included as a recommended therapy in the 2017 ACC/AHA or 2018 ESC guidelines [3,4].

This systematic review and meta-analysis evaluate the impact of structured exercise interventions on syncope recurrence, QoL, and physiological parameters in patients with VVS. Due to absence of formal guideline recommendations, this study aims to clarify the role of exercise-based strategies in the contemporary management of recurrent VVS.

## 2 Methods

### 2.1 Protocol and registration

This systematic review and meta-analysis were conducted in accordance with the guidelines outlined in the Preferred Reporting Items for Systematic Reviews and Meta-analyses (PRISMA) framework. The protocol was prospectively registered with the PROSPERO International Register of Systematic Reviews (CRD420251009841) [7]. Any deviations from the registered protocol are detailed in the Online Supplement; these modifications did not alter the predefined primary outcome or materially affect the overall conclusions of the review.

### 2.2 Search strategy and data sources

A comprehensive search strategy was developed in collaboration with an experienced medical librarian, incorporating the terms related to exercise interventions (cardiac rehabilitation OR exercise OR aerobic training OR yoga OR physical activity) and syncope (vasovagal syncope OR neurocardiogenic syncope OR reflex syncope OR orthostatic intolerance) combined with Boolean operators. The search was conducted across Ovid MEDLINE, Embase, and Emcare, for studies published between 1 January 1998 and 25 July 2025. The detailed search strategy is provided in the supplementary material.

Search results were imported into Covidence software for systematic screening. Titles and abstracts were independently reviewed by two authors (AE and CR). Full-text review of potentially eligible studies was also independently performed by the same two reviewers (AE and CR), with discrepancies resolved through discussion with senior authors (MA and HC).

### 2.3 Eligibility criteria

Studies published between 1 January 1998 and 25 July 2025 were eligible if they enrolled patients diagnosed with VVS or recurrent syncope of autonomic or reflex-mediated origin. Patients with syncope due to other mechanisms, including cardiac arrhythmia, structural heart disease, neurological disorders (e.g. epilepsy), or medication-induced syncope, were excluded.

Eligible interventions included structured exercise programs focused on aerobic or autonomic-modulating modalities, such as endurance or aerobic training, yoga, general physical exercise, or cardiac rehabilitation. To ensure physiologic relevance, we included only programs designed to influence autonomic or reflex pathways implicated in VVS. Accordingly, yoga interventions were required to incorporate structured physical components, such as postural sequences and graded cardiovascular activation, rather than meditation-only or breathing-focused formats. Interventions whose primary effects were limited to musculoskeletal conditioning, such as strength-only or resistance-based programs, were excluded, as were non-exercise regimens (e.g. orthostatic training) that do not target cardiovascular autonomic mechanisms.

Randomized controlled trials, open interventional, and prospective observational studies were considered if they reported relevant syncope outcomes. Abstracts, conference proceedings, case reports, reviews, and non-English publications were excluded. For overlapping patient cohorts, only the study with the largest sample size or most complete dataset were included to maintain data integrity and avoid duplications.

### 2.4 Data extraction

Two reviewers (AE and CR) independently extracted data using standardized Excel spreadsheet. Variables included study author, publication year, treatment period, geographic locations, patient demographics (age, sex), intervention type (type of physical activity), baseline syncope data, and diagnostic criteria. Outcome measures included syncope recurrence data, QoL measures, and physiological outcomes. Discrepancies between the reviewers were resolved through discussion with senior authors and co-authors.

### 2.5 Risk of bias assessment

The risk of bias in randomized controlled trials was assessed using the Cochrane Collaboration Risk of Bias tool, which evaluates domains including randomization, adherence, and outcome reporting [8]. The studies were further allocated to high, low, or unclear risk of bias. To assess the quality of the included non-randomized studies, the Newcastle-Ottawa Scale (NOS) was used, with ratings categorized as good quality, fair quality, and poor quality based on the Agency for Healthcare Research and Quality (AHRQ) standards [[9, [10], [11]]. Two investigators (AE and CR) independently performed all risk of bias and study quality assessments, evaluating randomized trials across the specified Cochrane domains and non-randomized studies on selection, comparability, and outcome domains, and documenting justification for each judgment made. Any disagreements were resolved by consensus with the senior authors (MA and HC).

### 2.6 Outcome measures

The primary outcome of interest was the recurrence of VVS, defined as the proportion of patients experiencing at least one syncopal episode during follow-up or the mean number of syncopal events per patient. Secondary outcomes included changes in QoL, assessed using validated measures such as the Syncope Functional Status Questionnaire (SFSQ), as well as physiological parameters, including heart rate (HR) and blood pressure (BP) changes.

### 2.7 Statistical analysis

In studies without a protocol-defined control arm, comparator data were taken from internal reference groups, consisting of participants who did not undertake structured exercise, while in other studies, pre-intervention baselines were used as within-cohort comparators. For studies reporting categorical recurrence over a fixed follow-up duration, an equivalent monthly event rate was derived by dividing recurrence counts by total follow-up time to permit time-standardized comparison across studies with different follow-up periods.

A random-effects model was employed to account for expected heterogeneity across studies, given variations in study design, intervention type, follow-up duration, and outcome assessment. Pooled proportions of syncope recurrence were calculated separately for control and intervention arms (Fig. 2A and 2B). For comparative analysis, the pooled mean difference (MD) in the number of VVS events was estimated using the restricted maximum likelihood (REML) method (Fig. 2C). Effect sizes were reported with a 95 % confidence interval.

Statistical heterogeneity was assessed using the I^2^ statistic, with τ^2^ and p-values also reported. Values of I^2^ > 50 % were considered indicative of substantial heterogeneity. Due to variability in outcome measures, secondary outcomes such as QoL, HR, and BP were summarized descriptively. Publication bias was not formally assessed, as fewer than ten studies were included in accordance with Cochrane recommendations [8]. Two-tailed p-values < 0.05 were considered statistically significant. All analyses were performed using R (V4.3.3, 2024–02–29).

## 3 Results

### 3.1 Study identification and selection

**Fig. 1:**
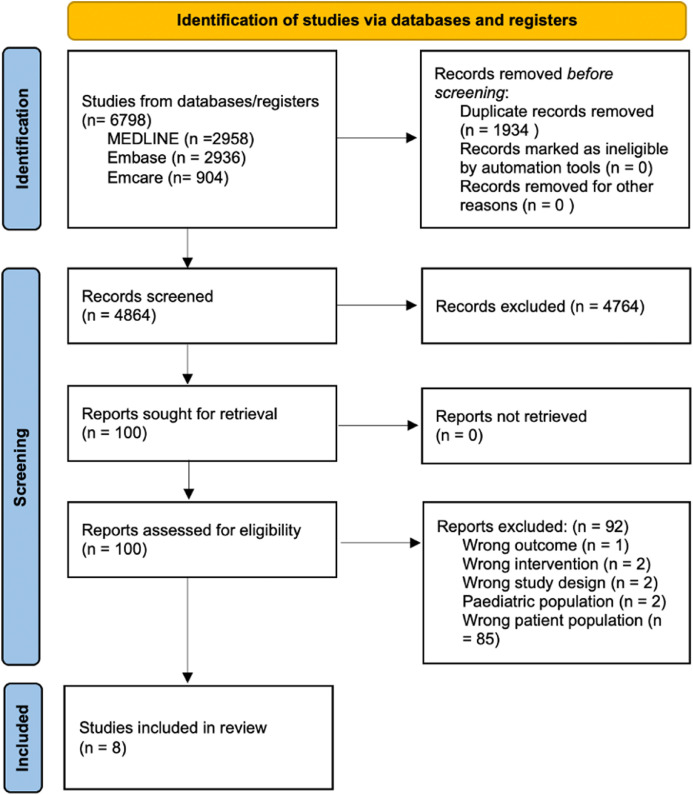
Flowchart of study inclusion pathway according to the Preferred Reporting Items for Systematic Reviews and Meta-Analyses (PRISMA).

We identified 6798 records (MEDLINE = 2958, Embase = 2936, Emcare = 904). After removing 1934 duplicates, 4864 records were screened and 4764 were excluded. All 100 full-text articles were retrieved and assessed; 92 were excluded (wrong outcome = 1, intervention = 2, study design = 2, pediatric population = 2, patient population = 85). Eight studies met the inclusion criteria and were included in the qualitative and quantitative analysis (Fig. 1).

### 3.2 Study characteristics

The characteristics of the eligible studies are summarized in Table 1. A total of eight studies were included: five randomized controlled trials (RCTs) [5,6,[15], [16], [17]], two interventional pilot trials [12,14], and one prospective cohort [13]. Studies were conducted across six countries, including the United Kingdom, Brazil, Netherlands, USA, India, and Iran between 1998 and 2022.

**Table 1:** Baseline study characteristics.

Author, Year published	Country	Study design	Total population	Age*	Mean follow-up (Months)	Female No.( %)	Baseline syncope frequency (episodes/month)	Vasovagal syncope diagnostic criteria
Mitinangi, 1998	United Kingdom	Open interventional	14	38 ± 11	-	75	0.33	History,head-up tilt test, lower-body suction protocol, absence of other disease
Gardenghi, 2007	Brazil	Randomized controlled trial	54	24.9 ± 3.35	4	59.3	0.33	History,head-up tilt test,structural and neurological causes excluded
Romme, 2010	Netherlands	Prospectiveobservational	100	38 ± 14	12	66	0.25	History,head-up tilt test, absence of other identifiable causes
Takahagi, 2014	Brazil	Randomized controlled trial	21	29 ± 9.54	3	85.7	0.46	History,head-up tilt test, exclusion of other cardiac/neurological causes
Gunda, 2015	United States	Open interventional pilot	44	21.52 ± 3.04	12	93.2	1.27	History, head-up tilt test, absenceof secondary causes
Shenthar, 2021	India	Randomized controlled trial	97	33.1 ± 16.6	14.3 ± 2.1	58.8	0.53	History, head-up tilt test, exclusion of other causes
Sharma, 2022	India	Randomized controlled trial	55	39.3 ± 15.04	12	65.5	0.87	History, head-up tilt test, absenceof other disease
Aghajani, 2022	Iran	Randomized controlled trial	50	34.5 ± 14.8	12	64	0.22	History, exclusion of alternative causes

*: Age in mean and standard deviation.

The combined sample size was 435 participants, with a mean age ranging from 21 to 39 years and 59 % female. The follow-up period varied between 2 and 18 months. At baseline, participants experienced between 1 and 4 syncopal episodes per month (mean ≈ 2.8 ± 1.9 episodes).

Across studies, diagnosis of VVS was based on characteristic clinical history (typical prodrome and triggers) and confirmed by head-up-tilt-table (HUTT) testing in seven of eight trials. One study used clinical criteria consistent with the European Society of Cardiology (ESC) guidelines [4].

### 3.3 Intervention details

The nature and intensity of exercise interventions varied in type and structure but shared a common focus on enhancing autonomic stability and cardiovascular conditioning (Table 2). Aerobic programs emphasised sustained cardiovascular training through treadmill or cycle ergometer exercise [5,6,17], while yoga-based interventions in addition to aerobic training also combined postural sequences, breathing control, and mindfulness techniques designed to influence autonomic tone [[14], [15], [16]]. Mixed regimens integrated stretching, resistance, and light endurance exercises to improve overall fitness and potentially influence circulatory reflexes [12,13]. Some included studies delivered additional behavioural therapies alongside exercise, for example, Romme et al. incorporated lifestyle counselling and counterpressure manoeuvres, while Aghajani et al. combined exercise with tilt-training [13,17]. Control groups generally received standard education and lifestyle guidance, including hydration, salt intake, avoidance of triggers, and counter-pressure manoeuvres without structured exercise.

**Table 2:** Intervention description and syncope recurrence data.

Author, Year published	Type of exercise regimen	Treatment period	Intervention description	Intervention duration & frequency	Intervention supervision	Control group	Syncope recurrence data						P value (Mean syncopal episodes: Intervention vs control)
							Intervention group			Control group			
							Total No.	Mean syncopal episode*	Recurrence No ( %)	Total No.	Mean syncopal episode*	Recurrence No ( %)	
Mitinangi, 1998	Physical exercise	Until target/ability (∼ 2 months)	Canadian Air Force 5BX/XBX	11–12 min, daily	Semi-supervised	-	14	0.14 ± 0.36	2 (14.3)	-	-	-	-
Gardenghi, 2007	Physical exercise	4 months	Stretching, cycling, strength training	60 min, 3 times/week	Supervised	Tilt training, pharmacological therapy, standard care	11	0.045 ± 0.10	2 (18.8)	43	0.048 ± 0.098	8 (18.6)	<0.94
Romme, 2010	Physical exercise	18 months	Physical exercise & standard care	Several times/week	Unsupervised	-	100	0.1 ± 0.22	4 (4.0)	-	-	-	<0.001
Takahagi, 2014	Aerobic training	3 months	Moderate-intensity cycle ergometer	35 mins, 4 times/week	Semi-supervised	Light exercise: 30-min walk/stretch	11	0 ± 0.2	1 (9.1)	10	0.67 ± 0.67	3 (23.3)	<0.01
Gunda, 2015	Yoga	3 months	Self-directed yoga sessions	60 mins, 3 times/week	Unsupervised	Standard care	21	0.43 ± 0.23	-	23	1.1 ± 0.17	-	<0.0001
Shenthar, 2021	Yoga	12 months	Guided yoga therapy	60 mins, 5 days/week	Semi-supervised	Standard care	50	0.09 ± 0.07	3 (6.0)	47	0.32 ± 0.27	17 (36.2)	<0.0001
Sharma, 2022	Yoga	12 months	Guided yoga therapy	40 mins, 5 sessions/week	Semi-supervised	Standard care	30	0.058 ± 0.058	1 (4.73)	25	0.21 ± 0.16	2 (7)	<0.0001
Aghajani, 2022	Physical exercise	12 months	Moderate-intensity aerobic exercise, rowing & cycling	45 min, 3x first month, then biweekly	Semi-supervised	Standard care, tilt-training	25	0.023 ± 0.044	1 (2.71)	25	0.053 ± 0.067	2 (5.22)	<0.05

Mean syncopal episode: average syncopal episode per month in each study population ± standard deviation.

The treatment period across studies ranged from 8 weeks to 12 months, with follow-up durations extending up to 18 months. Exercise sessions were conducted three to five times per week, lasting 30–60 min per session, depending on the intervention type. Supervision intensity varied, with some studies implementing fully supervised hospital-based rehabilitation sessions, whereas others utilised partially supervised or home-based programmes [5,6,[12], [13], [14], [15], [16], [17]]. Adherence, when reported, exceeded 80 % in six of eight studies, indicating good feasibility and participant compliance [5,6,[12], [13], [14], [15], [16], [17]].

### 3.4 Assessments of bias and study quality

All RCTs were assessed using the Cochrane Collaboration Risk of Bias tool and showed overall low risk in randomization, attrition, and reporting domains (Table 4) [8]. Performance bias was inevitably high, as participant blinding was not feasible for exercise interventions, while detection bias remained unclear in several trials due to unreported assessor masking. Among non-randomized studies, methodological quality was good, with NOS between 6 and 8 (Table 5) [[12], [13], [14]]. Overall, the included studies demonstrated acceptable methodological rigor, although limited blinding introduces minor performance and detection bias that should be considered when interpreting pooled results.

### 3.5 Primary outcome: Syncope recurrence

**Fig. 2:**
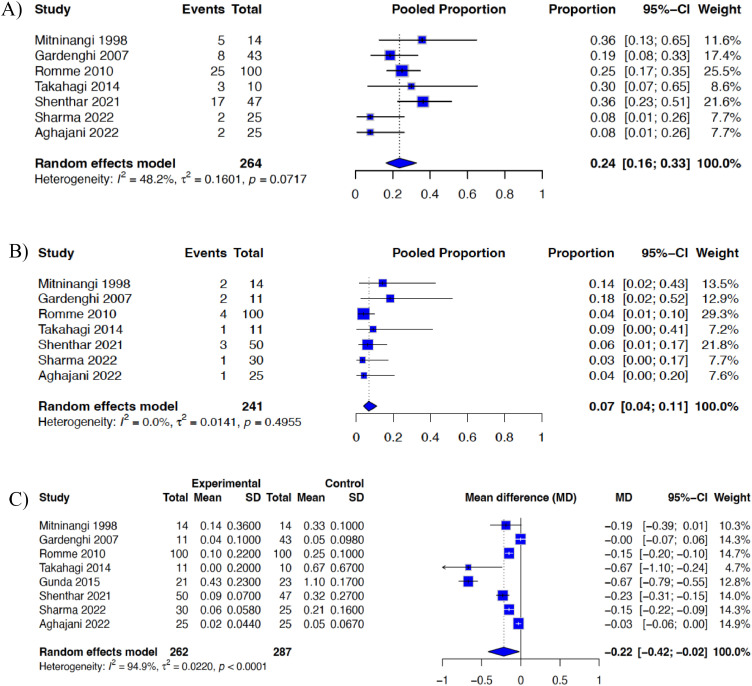
A) Meta-analytic pooled estimate of vasovagal syncope recurrence in the control group B) Meta-analytic pooled estimate of vasovagal syncope recurrence in the intervention group. C) Forrest plot of the pooled mean difference in vasovagal syncope events between intervention and control groups.

Across all eight studies, the rate of VVS recurrence was lower in the exercise intervention groups compared with control or standard care groups (Fig. 2). In the control group (Fig. 2A), the pooled recurrence proportion was 0.24 (95 % CI 0.16–0.33), indicating that approximately one in four participants experienced a recurrent syncopal episode despite standard management. In contrast, the intervention groups (Fig. 2B) demonstrated a significantly lower pooled recurrence proportion of 0.07 (95 % CI 0.04–0.11).

This corresponded to an absolute reduction of roughly 17 % in recurrence risk and a pooled mean difference of −0.22 (95 % CI −0.42 to −0.02) in favour of exercise interventions (Fig. 2C). Heterogeneity was moderate for control group data (I^2^ = 48.2 %), negligible for intervention groups (I^2^ = 0 %), and high for mean difference analysis (I^2^ = 94.9 %), reflecting the variation in intervention duration and outcome definitions across studies. Descriptively, yoga-based interventions were associated with lower recurrence rates compared to those without such exercises. Despite the methodological diversity, all studies demonstrated a consistent trend favouring exercise versus the standard of care.

### 3.6 Secondary outcomes

#### 3.6.1 Quality of life

Four studies assessed the quality of life using SFSQ [[13], [14], [15], [16]]. All demonstrated significant improvement following intervention, with mean reductions in SFSQ scores of 30–45 points (*p* < 0.001), indicating fewer functional limitations and improved confidence. The most pronounced effects were seen in yoga-based programs [[14], [15], [16]], while Romme et al.,. 2010 reported comparable benefits at six months (*p* < 0.0001) [13]. Collectively, these findings highlight that structured exercise enhances both physical functioning and psychological resilience in patients with recurrent VVS.

#### 3.6.2 Heart rate and blood pressure

Five studies evaluated cardiovascular parameters (Table 3) [5,6,12,14,16]. Only two studies reported significant reductions in resting HR (6–8 bpm, *p* < 0.01) [5,12], consistent with improved vagal tone and autonomic function. The remaining studies found no significant change (*p* > 0.05). Similarly, a significant BP reduction was observed only in one study (*p* < 0.0001) [12], while the other study reported minimal variation. Overall, exercise training appears to promote autonomic reconditioning rather than major hemodynamic shifts, with modest improvements in cardiovascular efficiency potentially contributing to lower recurrence rates.

**Table 3:** Secondary outcomes.

Author, Year published	Quality of life change (SFSQ)			Heart rate (mean bpm)			Blood Pressure (mean mmHg)*		
	Pre-intervention	Post-intervention	P value	Pre-intervention	Post-intervention	P value	Pre-intervention	Post-intervention	P value
Mitinangi, 1998	-	-	-	71.4 ± 2.5	64.7 ± 2.2	<0.0001	109/62 ± 1.8/1.8	104/56 ± 1.6/1.4	<0.0001
Gardenghi, 2007	-	-	-	69 ± 9	60 ± 8	<0.01	-	-	-
Romme, 2010	34	22	<0.001	-	-	-	-	-	-
Takahagi, 2014	-	-	-	64 ± 8	66 ± 10	>0.05	-	-	-
Gunda, 2015	67± 7.8	29.8 ± 4.6	<0.001	79 ± 6	74 ± 4	>0.05	120/74 ± 8/3	115/69 ± 3/5	>0.05
Shenthar, 2021	31.4 ± 7.2	22.2 ± 4.7	<0.001	-	-	-	-	-	-
Sharma, 2022	49.90 ± 25.90	22.06 ± 22.74	<0.001	71± 12.59	77 ± 11.09	0.128	-	-	-
Aghajani, 2022	-	-	-	-	-	-	-	-	-

SFSQ: Syncope Functional Status Questionnaire average score ± standard deviation.

BPM: Beats per minute average ± standard deviation.

*= Systolic blood pressure/diastolic blood pressure average ± standard deviation.

**Table 4:** Risk of bias assessment (Cochrane Collaboration Risk of Bias Tool).

**Table 5:** Risk of bias assessment (Newcastle-Ottawa Scale).

Studies	Selection				Comparability	Exposure			NOS quality score	AHRQ standards
	Representativeness of exposed cohort	Selection of non-exposed cohort	Ascertainment of exposure	Outcome not present before the exposure	Comparability of cases and control on the basis of the design or analysis	Assessment of outcome	Was follow-up long enough for outcomes to occur	Adequacy of follow-up of cohorts		
Mitinangi et al., 1998	∗	*	*	*	*	*	*		7	Good
Romme et al., 2010	*	*	*	*	*	*	*	*	8	Good
Gunda et al., 2015	*	*	*	*	*	*	*	*	8	Good

Thresholds for converting the Newcastle–Ottawa scales to the Agency for Healthcare Research and Quality (AHRQ) standards (good, fair, and poor):(10).

Good quality: 3 or 4 stars in selection domain and 1 or 2 stars in comparability domain and 2 or 3 stars in outcome/exposure domain.

Fair quality: 2 stars in selection domain and 1 or 2 stars in comparability domain and 2 or 3 stars in outcome/exposure domain.

Poor quality: 0 or 1 star in selection domain or 0 star in comparability domain or 0 or 1 star in outcome/exposure domain.

## 4 Discussion

This meta-analysis establishes that exercise, encompassing both traditional endurance training and yoga-based modalities, is associated with lower VVS recurrence and enhances quality of life. Across eight studies including 435 participants, the rate of recurrent syncope was lower in the intervention groups (7 %; 95 % CI 0.04–0.11) compared with the control groups (24 %; 95 % CI 0.16–0.33), representing an absolute reduction of approximately 17 % in recurrence risk and a pooled mean difference of –0.22 (95 % CI −0.42 to −0.02). These findings support consideration of structured exercise as an adjunct to standard measures such as education, hydration, and counterpressure manoeuvres recommended by the 2018 ESC and 2017 ACC/AHA guidelines [3,4].

From a cardiac perspective, vasovagal syncope reflects a biphasic autonomic sequence. Initial orthostatic stress produces a rise in sympathetic outflow and vigorous contraction of a relatively underfilled left ventricle. Classic experimental work by Victor et al. and the mechanistic analysis of Furlan et al. show that this combination activates mechanosensitive ventricular C-fiber afferents, which then trigger a centrally mediated shift toward abrupt sympathetic withdrawal and enhanced vagal efferent discharge [24,25]. The resulting bradycardia, vasodilation, and fall in cardiac output account for the final hypotensive-bradycardic phase of VVS [3,19]. Regular exercise may reduce susceptibility to this reflex by increasing venous return, improving baroreflex sensitivity, and attenuating the magnitude of sympathetic surges that initiate the C-fiberactivation [4,20]. Exercise training has also been associated with a more stable autonomic profile, characterised by improved vagal-sympathetic balance and reduced oscillatory autonomic variability, without predisposing to exaggerated vagal reflexes during orthostatic stress [5,6,19,20]. These adaptations provide a biologically plausible framework that may help lower recurrence rates observed across the included trials. However, the included studies did not directly evaluate these mechanistic pathways, and the proposed explanations remain consistent with established Bezold-Jarish physiology [18].

Exercise has been associated with central neuroplastic adaptations that may enhance autonomic regulation. Functional and neurophysiological studies suggest that endurance training can strengthen connectivity between the insula, ventrolateral medulla, and nucleus tractus solitarius, key centres for baroreflex and cardiovascular integration [[20], [21], [22]]. These adaptations have been suggested in experimental studies to improve synchronisation between cortical and brainstem autonomic circuits, potentially supporting faster recovery and greater stability during orthostatic or exertional stress [[20], [21], [22]]. Exercise may also increase efficiency within the central autonomic network, reducing sympathetic latency and improving reflex precision, which could enhance circulatory control and tolerance to preload shifts [[20], [21], [22]].

Although the included trials varied in design and were unblinded, this reflects the practical constraints of evaluating behavioural and exercise-based interventions, where participant blinding is difficult to control. Importantly, despite the variability, all studies demonstrated a consistent direction of effect, with lower syncope recurrence observed in the intervention groups. Syncope is an episodic and variably expressed outcome; the reproducibility of benefit across multiple independent cohorts demonstrates that observed improvements are unlikely to be explained by expectancy effects alone.

Several included studies incorporated additional lifestyle components alongside exercise, such as counterpressure manoeuvres or tilt-training [13,17]. This multimodal design reflects clinical practice, where exercise is rarely driven in isolation, while such co-interventions prevent attribution of effect to exercise alone within individual trials, the consistent lower recurrence observed across studies, including those in which exercise was the principal active component, supports a contribution of exercise to overall autonomic stabilisation.

The magnitude of benefit observed here compares favourably with pharmacological therapy. Fludrocortisone and midodrine provide only modest hemodynamic support without addressing the neural instability driving reflex syncope, while beta blockers remain inconsistent and poorly tolerated in younger patient [3,4,[14], [15], [16]]. Exercise training may represent a complementary strategy aimed at modulating autonomic responses, potentially targeting neural reflex instability through physiological conditioning rather than pharmacologic suppression. As a non-invasive intervention, structured exercise could reduce reliance on chronic medication or device-based therapies in selected patients, although hypothesis requires confirmation in larger prospective studies.

While resting HR and BP showed only small, non-significant changes in most trials, the potential benefit of exercise may lie in improved reflex buffering capacity, which is a reduction in the amplitude and latency of autonomic oscillations that produce abrupt vagal surges. These neural and sinus node-level adaptations may be especially important in patients with cardioinhibitory phenotypes, where exercise-mediated remodelling of vagal reflexes could attenuate sinus pause duration or abort bradycardic sequences before syncope onset [17,19,20].

Importantly, improved QoL and psychological resilience were consistently observed, reflecting both physiologic and cognitive-autonomic integration. By restoring patient confidence and reducing anticipatory anxiety, exercise likely interrupts the cyclical reinforcement of syncope susceptibility mediated by cortical-autonomic feedback loops [2,16,20]. Similar psychosomatic benefits have been documented in cardiac rehabilitation populations, supporting the bidirectional relationship between autonomic tone and mental state [16,20].

Taken together, the findings of this meta-analysis suggest that exercise is associated with lower VVS recurrence and may enhance autonomic stability, though several limitations should be acknowledged. The included studies were small, single-centre trials with variation in exercise modality, supervision intensity, and follow-up duration, contributing to heterogeneity and limiting generalisability. This also prevented separate analysis of aerobic vs yoga interventions, despite their shared autonomic conditioning features. Although methodological quality was acceptable, the behavioural nature of exercise interventions precluded blinding, introducing inherent risks of performance and detection bias. Follow-up periods were relatively short, making it uncertain whether benefits persist long-term. Importantly, despite the hypothesised autonomic mechanisms underlying improvement, objective autonomic outcomes such as HRV indices, baroreflex sensitivity or catecholamine measures were rarely assessed. This limits the ability to confirm whether autonomic reconditioning directly mediated the reduction in syncope. Continuous rhythm or electrophysiologic monitoring was seldom used, reflecting real-world clinical practice where such tools are reserved for complex or pacing candidates.

Variability in diagnostic criteria, adherence reporting, and outcome definitions further complicates cross-study comparisons. Future multicentre randomized trials should employ standardised protocols, isolated exercise assessments, longer follow-up, and harmonised autonomic and QoL metrics to better define the clinical durability and mechanistic pathways of benefit. Pragmatic use of ambulatory monitoring and autonomic profiling could clarify how structured exercise reconditions reflex control and stabilises cardiovascular function, ultimately supporting its broader integration into syncope management frameworks.

## 5 Conclusion

This systematic review and meta-analysis suggests that structured exercise is associated with lower VVS recurrence and potentially improves autonomic function. Exercise lowered recurrence by about 20 % compared with standard care, alongside consistent improvements in QoL scores.

## Data availability statement

The data underlying this article are available in the article and its online supplementary material.

## Disclosures and relationships with industry

The authors report no relationships with industry and no disclosures relevant to the contents of this manuscript.

## Funding

This research received no specific grant from any funding agency in the public, commercial, or not-for-profit sectors.

## Ethics approval (human participants)

Not applicable. This study is a systematic review and meta-analysis of published literature and did not involve direct interaction with human participants or access to identifiable private data; therefore, institutional review board/ethics committee approval was not required.

## CRediT authorship contribution statement

**Amin Esmailian:** Writing – original draft, Methodology, Investigation, Formal analysis, Data curation, Conceptualization. **Cyrus Raki:** Investigation, Formal analysis, Data curation. **Hui Chen Han:** Writing – review & editing, Validation, Supervision. **Mohammad Alasti:** Writing – review & editing, Validation, Supervision, Conceptualization.

## Declaration of competing interest

The authors declare no conflict of interest.

## Appendix Supplementary materials

Image, application 1

## References

[1] Ganzeboom K.S., Mairuhu G., Reitsma J.B., Linzer M., Wieling W., van Dijk N. (2006). Lifetime cumulative incidence of syncope in the general population: a study of 549 Dutch subjects aged 35–60 years. J Cardiovasc Electrophysiol.

[2] Ng J., Sheldon R.S., Maxey C., Ritchie D., Raj V., Exner D.V. (2019). Quality of life improves in vasovagal syncope patients after clinical trial enrollment regardless of fainting in follow-up. Auton Neurosci: Basic Clin.

[3] Shen W.K., Sheldon R.S., Benditt D.G., Cohen M.I., Forman D.E., Goldberger Z.D. (2017). 2017 ACC/AHA/HRS guideline for the evaluation and management of patients with syncope. Circulation.

[4] Brignole M., Moya A., de Lange F.J., Deharo J.C., Elliott P.M., Fanciulli A. (2018). 2018 ESC guidelines for the diagnosis and management of syncope. Eur Heart J.

[5] Gardenghi G., Rondon M.U.P.B., Braga A.M.F.W., Scanavacca M.I., Negrao C.E., Sosa E. (2007). The effects of exercise training on arterial baroreflex sensitivity in neurally mediated syncope patients. Eur Heart J.

[6] Takahagi V.C.M., Costa D.C., Crescêncio J.C., Gallo L. (2014). Physical training as non-pharmacological treatment of neurocardiogenic syncope. Arq Bras Cardiol.

[7] Page M.J., McKenzie J.E., Bossuyt P.M., Boutron I., Hoffmann T.C., Mulrow C.D. (2021). The PRISMA 2020 statement: an updated guideline for reporting systematic reviews. BMJ.

[8] Sterne J.A.C., Savović J., Page M.J., Elbers R.G., Blencowe N.S., Boutron I. (2019). RoB 2: a revised tool for assessing risk of bias in randomisedtrials. BMJ.

[bib0009] Wells G.A., Shea B., O’Connell D., Peterson J., Welch V., Losos M., Tugwell P. (2020). http://www.ohri.ca/programs/clinical_epidemiology/oxford.asp.

[10] Agency for Healthcare Research and Quality (AHRQ) (2014). https://effectivehealthcare.ahrq.gov/products/cer-methods-guide/overview.

[11] Stang A. (2010). Critical evaluation of the Newcastle–Ottawa Scale for the assessment of the quality of nonrandomized studies in meta-analyses. Eur J Epidemiol.

[12] Mitinangi B.L., Hainsworth R. (1998). Increased orthostatic tolerance following moderate exercise training in patients with unexplained syncope. Heart (Br. Card. Soc..

[13] Romme J J C M, van Dijk N., Boer K.R., Dekker L.R., Stam J., Reitsma J.B., Wieling W. (2010). Prospective evaluation of non-pharmacological treatment in vasovagal syncope: the Physical Counterpressure Manoeuvres Trial II. EP Eur.

[14] Gunda S., Bhansali S., Kumar P., Pillarisetti J., Lakkireddy D. (2015). Role of yoga as an adjunctive therapy in patients with neurocardiogenic syncope: a pilot study. J Interv Electrophysiol.

[15] Shenthar J., Gangwar R.S., Banavalikar B., Benditt D.G., Lakkireddy D., Padmanabhan D. (2021). A randomized study of yoga therapy for the prevention of recurrent reflex vasovagal syncope. EP Eur.

[16] Sharma G., Ramakumar V., Sharique M., Bhatia R., Naik N., Mohanty S. (2022). Effect of yoga on clinical outcomes and quality of life in patients with vasovagal syncope (LIVE-Yoga). JACC Clin Electrophysiol.

[17] Aghajani F., Tavolinejad H., Sadeghian S., Bozorgi A. (2022). Implementation of supervised physical training to reduce vasovagal syncope recurrence: a randomized control trail. J Cardiovasc Electrophysiol.

[18] Mark A.L. (1983). The Bezold-Jarisch reflex revisited: clinical implications of inhibitory reflexes originating in the heart. J Am Coll Cardiol.

[19] Van Dijk J.G., Van Rossum I.A., Thijs R.D. (2021). The pathophysiology of vasovagal syncope: novel insights. Auton Neurosci.

[20] Besnier F., Labrunée M., Pathak A., Pavy-Le Traon A., Galès C., Senard J.M. (2017). Exercise training-induced modifications in autonomic nervous system: an update for cardiac patients. Ann Phys Rehabil Med.

[21] Williamson J.W., McColl R., Mathews D. (2003). Evidence for central command activation of human insular cortex during exercise. J Appl Physiol.

[22] De la Cruz F., Geisler M., Schumann A., Herbsleb M., Kikinis Z., Weiss T. (2022). Central autonomic network alterations in male endurance athletes. Sci Rep.

[23] Abu-Ghazaleh D., Taylor D.A., Roberts L., Singh I., Cruzat V., Rose’Meyer R.B (2025). The pathophysiology of Vasovagal syncope and new approaches to its pharmacological treatment. J Cardiovasc Pharmacol Ther.

[24] Furlan R., Piazza S., Dell’Orto S., Gentile E., Cerutti S., Pagani M. (1993). Early and late effects of exercise and athletic training on neural mechanisms controlling heart rate. Cardiovasc Res.

[25] Victor R.G., Thorén P., Morgan D.A., Mark A.L. (1989). Differential control of adrenal and renal sympathetic nerve activity during hemorrhagic hypotension in rats. Circ Res.

