# Supplementary material for: The role of exercise in reducing vasovagal syncope recurrence: A systematic review and meta-analysis
Source: Am J Prev Cardiol. 2026 Feb 26;26:101504. doi: 10.1016/j.ajpc.2026.101504 (PMC13084098; doi:10.1016/j.ajpc.2026.101504)
Supplement: Supplementary file 1 [file mmc1.docx]

**Supplementary Appendix- Deviations from the Registered PROSPERO Protocol (CRD420251009841)**

The review was conducted in accordance with the registered PROSPERO protocol (CRD420251009841). The following methodological refinements occurred during the conduct of the review:

**1. Data synthesis approach**

The protocol initially specified narrative synthesis due to anticipated heterogeneity in exercise modalities and outcome reporting. Following study identification and data extraction, sufficient methodological similarity across included studies allowed quantitative pooling. A random-effects meta-analysis was therefore performed to provide pooled effect estimates for syncope recurrence.

**2. Study design inclusion**

The protocol specified inclusion of randomized studies only. During screening, prospective interventional and observational studies meeting eligibility criteria were also included to comprehensively capture available evidence in this emerging field, given the limited number of randomized trials.

**3. Exercise supervision criteria**

The protocol restricted inclusion to supervised cardiac exercise programs. In practice, studies incorporating structured home-based or partially supervised programs were included where exercise regimens were predefined and monitored, as these reflected real-world implementation of structured exercise therapy.

**4. Search date restriction**

The protocol indicated no search date restrictions. The final search was limited to studies published between 1 January 1998 and 25 July 2025 to ensure consistency with contemporary diagnostic criteria and modern exercise methodologies.

**5. Certainty of evidence assessment**

The protocol proposed use of the GRADE framework with a Summary of Findings table. A formal GRADE assessment was not performed due to heterogeneity in study design and outcome reporting across included trials. Risk of bias was assessed at the study level using the Cochrane Risk of Bias tool for randomized trials and the Newcastle–Ottawa Scale for non-randomized studies.

These modifications represent methodological refinements made during review conduct. The predefined primary outcome of syncope recurrence was not altered, and these deviations did not materially affect the overall conclusions of the study.
